# Production of functional human galectin-1 in transplastomic tobacco and simplified recovery via batch-mode purification

**DOI:** 10.3389/fpls.2025.1721928

**Published:** 2026-01-02

**Authors:** Catalina Francisca Vater, Juan Manuel Pérez Sáez, Juan Carlos Stupirski, Mora Massaro, Federico Gabriel Mirkin, Fernando Félix Bravo-Almonacid, Gabriel Adrián Rabinovich, Mauro Miguel Morgenfeld

**Affiliations:** 1 Laboratorio de Biotecnología Vegetal, Instituto de Investigaciones en Ingeniería Genética y Biología Molecular “Dr. Héctor N. Torres” (INGEBI-CONICET), Ciudad Autónoma de Buenos Aires, Argentina; 2 Facultad de Ciencias Exactas y Naturales, Universidad de Buenos Aires, Ciudad Autónoma de Buenos Aires, Argentina; 3 Laboratorio de Glicomedicina, Programa de Glicociencias, Instituto de Biología y Medicina Experimental (IBYME-CONICET), Ciudad Autónoma de Buenos Aires, Argentina; 4 Departamento de Ciencia y Tecnología, Universidad Nacional de Quilmes, Buenos Aires, Argentina; 5 Laboratory of Glycoimmunology, Caixa Research Institute, Barcelona, Spain

**Keywords:** batch mode purification, biopharmaceutical production, chloroplast transformation, human galectin-1, plant molecular farming, transplastomic tobacco

## Abstract

Plant molecular farming has established itself as a transformative technology for the cost-effective and sustainable production of biopharmaceuticals, offering scalable solutions to meet growing global demand. Among the different stable plant expression systems, plastid-based platforms are particularly attractive due to their high recombinant protein accumulation potential, genetic stability, and reduced risk of transgene escape. Human Galectin-1 (hGAL1) is a β-galactoside-binding lectin with potent immunomodulatory properties, positioning it as a promising therapeutic candidate for autoimmune and inflammatory diseases. Preserving its native conformation and carbohydrate-binding capacity is essential to keep its biological activity, and both properties may be compromised under suboptimal expression or purification conditions. Here, we demonstrate the relevance of chloroplast transformation in *Nicotiana tabacum* as a platform for producing functional hGAL1, which accumulated up to 5.67 mg per kg of leaf tissue, corresponding to ~0.05% of total soluble protein (TSP). Using a simplified batch-mode purification strategy, intact hGAL1 retaining carbohydrate-binding activity was obtained and functional properties as shown by its ability to induce T cell apoptosis in a dose-dependent manner. These results highlight the potential of a transplastomic tobacco platform to deliver biologically active human lectins with therapeutic relevance, while minimizing downstream processing complexity, supporting their use in cost-effective biopharmaceutical production.

## Introduction

Plant molecular farming (PMF) has emerged as a versatile and sustainable strategy for the production of high-value recombinant proteins. In some cases PMF offers certain advantages of scalability, reduced costs, and improved endotoxin-free biosafety compared to conventional microbial and mammalian cell systems (Burnett and Burnett, 2020; Buyel, 2019). At the moment there are many examples of human proteins expressed in plant cells that retain their activity indicating that folding occurs correctly as hIDO1, hFGF or hEGF (Bellucci et al., 2021; Wang et al., 2023; Müller et al., 2024). Moreover, this strategy has enabled the production of vaccines, therapeutic antibodies, and enzymes, some of which have functional folding reached clinical trials or market authorization (Schillberg and Finnern, 2021), including Elelyso^®^ and Elfabrio^®^ (Protalix Biotherapeutics) and Covifenz^®^ (Medicago). Within PMF platforms, chloroplast transformation, also known as transplastomic plants, stands out as a robust expression system due to the exceptional levels of protein accumulation achievable in plastids (Bock, 2015; Maliga and Bock, 2011; Daniell et al., 2021).

The plastid genome is highly polyploid with thousands of genome copies per cell (Shaver et al., 2006), which enables massive accumulation of heterologous proteins when transgenes are integrated by homologous recombination. Moreover, chloroplast transformation bypasses the gene silencing effects typical of nuclear transformation and supports polycistronic transcription units resembling operons (Scotti et al., 2013; Barkan and Goldschmidt-Clermont, 2000). Additional advantages include stable maternal inheritance that limits transgene flow via pollen and genetic stability across generations (Lal et al., 2020). As a result, heterologous protein accumulation in plastids has reached levels exceeding 70% of total soluble protein (Oey et al., 2009; Castiglia et al., 2016). Our group and others have contributed to expanding this field by developing plastid-based expression of antigens, growth factors, and therapeutic proteins in *Nicotiana tabacum* (Morgenfeld et al., 2020; Müller et al., 2024). These achievements highlight the enormous potential of transplastomic plants as factories for biopharmaceuticals.

Nevertheless, several challenges still constrain the industrial application of plastid biotechnology. Expression levels vary widely depending on the protein of interest, and reliable predictive rules for accumulation efficiency are still lacking (Ahmad et al., 2016). Furthermore, plastids lack glycosylation and other complex post-translational modifications, which can limit the expression of proteins requiring them for functionality (Lehtimäki et al., 2015). In contrast, proteins that are soluble, stabilized by disulfide bonds and non-glycosylated represent excellent candidates for plastid expression. Finally, downstream processing is considered the major economic bottleneck, often accounting for up to 80% of total manufacturing costs (Buyel, 2015). Traditional chromatography steps are expensive, time-consuming, and prone to clogging due to plant secondary metabolites, stressing the need for simplified purification strategies compatible with large-scale deployment (Buyel, 2024).

Human Galectin-1 (hGAL1) is a prototype member of the galectin family. It forms homodimers composed of ~14.5 kDa subunits, each carrying a conserved carbohydrate recognition domain (CRD) that preferentially binds to N-acetyllactosamine motifs (Troncoso et al., 2023; Porciúncula-González et al., 2021). hGAL1 is expressed in multiple immune and stromal cell compartments, where it regulates apoptosis, angiogenesis, and immune tolerance (Perillo et al., 1995; Rabinovich et al., 1997; Rabinovich et al., 1999; Rabinovich, 2005). Extensive studies have established its therapeutic relevance, demonstrating anti-inflammatory and immunomodulatory effects in diverse models of chronic inflammation, autoimmunity, and neurodegeneration. In fact, this lectin reduces disease severity in murine models of rheumatoid arthritis, colitis, diabetes, uveitis, multiple sclerosis, and Sjögren disease, largely by promoting apoptosis of activated T cells and skewing immune responses toward Th2 and regulatory T (Treg) cell profiles (Rabinovich et al., 1999; Perone et al., 2006; Santucci et al., 2003; Toscano et al., 2006; Starossom et al., 2012; Toscano et al., 2018; Martínez Allo et al., 2020; Morosi et al., 2021; Sundblad et al., 2021; Rabinovich et al., 2025). Additionally, hGAL1 shows neuroprotective effects after cerebral ischemia and alleviates atopic dermatitis in mice, highlighting its potential as a versatile therapeutic agent (Qu et al., 2011; Corrêa et al., 2017). Interestingly, recent studies demonstrated the ability of GAL1 to reprogram myeloid cells toward an immunosuppressive phenotype (Blidner et al., 2025), suggesting the ability of this lectin to control both lymphoid and myeloid cell compartments in a myriad of pathologic conditions.

Recombinant Human Galectin-1 has been produced as research reagent in several conventional heterologous expression systems. Bacterial expression in *Escherichia coli* is the most widely used approach, enabling high-yield production of recombinant hGAL1 that is properly folded and biologically active after purification (Rabinovich et al., 1999; Toscano et al., 2006). Yeast platforms, including *Pichia pastoris*, have also been explored for lectin production and offer advantages in secretion and scalability, although the hyperglycosylating nature of yeast can be incompatible with proteins—such as hGAL1—that require precise disulfide bond formation but do not undergo N-glycosylation. Mammalian cell expression (e.g., CHO or HEK293) provides native folding and post-translational processing and has been used to obtain recombinant galectins with immunomodulatory activity, but these systems are substantially more expensive, require complex infrastructure, and typically yield lower amounts of purified protein. In this context, plant-based platforms—and particularly plastid transformation—represent an attractive complementary strategy, combining the ability to fold cysteine-rich proteins in an oxidizing environment with agricultural scalability and inherent endotoxin-free biosafety. From a biochemical perspective, hGAL1 is a soluble protein stabilized by three intrachain disulfide bonds and does not require glycosylation for folding or activity (Guardia et al., 2014). This makes it particularly well-suited for plastid expression, as the chloroplast stroma supports oxidative folding and disulfide bond formation (Wittenberg and Danon, 2008).

In this work, we evaluated the potential of *N. tabacum* chloroplasts as a platform for hGAL1 production. Transplastomic lines were generated and confirmed to be homoplasmic, accumulating hGAL1 predominantly in the soluble protein fraction. To address downstream challenges, we implemented a simplified batch-mode lactosyl-Sepharose affinity capture. Compared with an equivalent column-based affinity protocol using the same resin, the batch mode purification procedure simplified the purification workflow increasing protein recovery, avoiding resin clogging and demonstrating scalability to larger biomasses. The enriched hGal1 fraction obtained was suitable for downstream functional assays Plant-derived hGAL1 preserved its carbohydrate-binding activity and induced apoptosis in Jurkat T cells, confirming its biological properties. Although the pro-apoptotic potency is lower than observed for bacterially-produced hGAL1, the results demonstrate that chloroplasts can produce functional lectins of therapeutic relevance.

Altogether, our findings provide proof-of-concept for the stable expression and simplified purification of biologically active hGAL1 in tobacco plastids. This approach addresses two central challenges of molecular farming: the production of correctly folded human proteins and the development of cost-efficient purification methods. Future research should focus on enhancing expression yields, exploring stabilizing formulations, and refining purification strategies to fully exploit the potential of chloroplast biotechnology for the scalable production of human lectins and other therapeutic proteins.

## Materials and methods

### Chloroplast transformation vector

**Figure 1:**
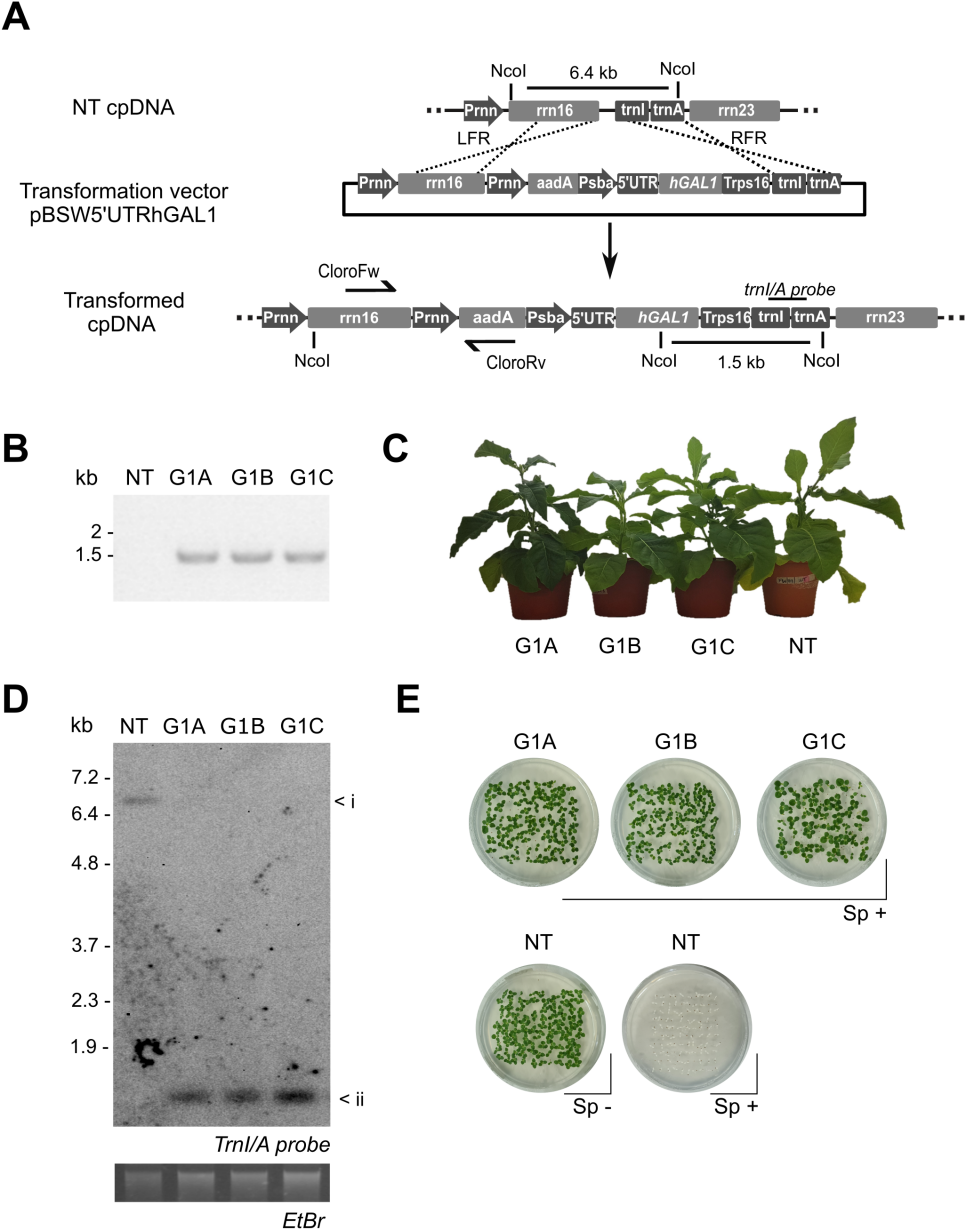
Generation and molecular characterization of transplastomic *Nicotiana tabacum* lines. **(A)** Schematic representation of the chloroplast transformation vector pBSW5’UTRhGAL1 (upper panel) and its targeted integration into the plastid genome (lower panel). The expression cassette comprises the *hGAL1* and the *aadA* selectable marker, flanked by homologous recombination sequences: the 3′ region of rrn16 (LFR) and the *trnI-trnA* intergenic region (RFR). Locations of PCR primers (CloroFw/Rv), *NcoI* restriction sites, and probe for Southern blot analysis are indicated. **(B)** PCR confirmation of transgene integration in primary regenerants (T0) corresponding to independent G1 A–C lines (expected amplicon size: 1.5 kb). **(C)** Phenotypic appearance of greenhouse-grown plants at 15 weeks post-germination. **(D)** Southern blot analysis of 1 µg NcoI-digested genomic DNA using a trnI–trnA probe. Expected fragment sizes: NT (6.5 kb), G1 A–C (1.5 kb). Ethidium bromide-stained gel is shown as a loading control. **(E)** Germination assay on selective medium. Upper panel: 100 T1 seeds from each transplastomic line (G1 A, G1 B, G1 C) germinated in MS with spectinomycin as selector agent (Sp^+^, 500 mg/L). Lower panel: 100 NT seeds were germinated in MS medium with or without spectinomycin (Sp^-^). Photo was taken after 7 days.

The human GAL1 coding sequence (NCBI #3956) was excised from pGem-hGAL1 and inserted into the plastid transformation vector pBSWUTR, previously developed in our laboratory (Wirth et al., 2006). Cloning was carried out using *NdeI* and *XbaI* restriction sites, and the construct was validated by Sanger sequencing. The expression cassette included the psbA promoter/5′UTR, the *aadA* selectable marker, and plastid recombination flanks corresponding to the rrn16 and trnI-trnA regions. The schematic representation of the pBSW5’UTRhGAL1 vector is shown in **Figure 1A**.

### Tobacco plastid transformation and plant regeneration

Plastid transformation was performed in *N. tabacum* cv. Petite Havana by biolistic delivery using a PDS-1000/He device (Bio-Rad), following adapted protocols previously reported (Maliga, 2004). Expanded leaves were bombarded with gold particles (0.6 μm, 50 μg) coated with 10 μg plasmid DNA under 1,100 psi helium pressure. Spectinomycin-resistant shoots were selected on RMOP medium (Svab et al., 1990) containing 500 mg/L spectinomycin and subsequently transferred to MS medium (Murashige and Skoog, 1962) containing 500 mg/L spectinomycin for root development. To achieve homoplasmy, putative transformants underwent three cycles of regeneration under selection before transfer to soil. Transgene integration was initially screened in primary regenerants (T0) by PCR using primers CloroFw (5′-GTATCTGGGGAATAAGCATCGG-3′) and CloroRv (5′-CGATGACGCCAACTACCTCTG-3′), which yield a 1,450 bp product.

### Plant cultivation

Seeds from the first progeny (T1) confirmed transplastomic lines were surface-sterilized (10% bleach or sodium hypochlorite vapor), germinated on MS medium supplemented with spectinomycin, and grown under a 16 h light/8 h dark photoperiod at 25°C. For greenhouse cultivation, plants were maintained at 20–25°C with a 16 h light cycle until 14–18 weeks of age, when leaves were harvested for analyses.

### Molecular analyses (southern and northern blot)

Blot analyses were performed using the DIG system (Roche) following the manufacturer’s instructions. Total DNA was isolated from T1 plants by the CTAB method (Allen et al., 2006) and digested with *NcoI* prior to electrophoresis and transfer to nylon membranes. 1 µg of digested DNA was loaded per lane. Hybridization with a DIG-labeled trnI/A probe was used to assess site-specific integration and homoplasmy. For transcript analysis, total RNA was purified from T1 plants with TRIzol (Invitrogen), separated on denaturing agarose gels (1 µg of total RNA per lane), and hybridized with DIG-labeled probes specific for *hGAL1* and *aadA*. Signals were visualized by chemiluminescence using CSPD as substrate.

### Protein extraction and western blot analysis

For total protein extraction, 50 mg of leaf tissue of T1 plants were ground directly in 200 µl of 1X Laemmli buffer (Laemmli, 1970) and boiled at 99°C for 10 min. For solubility analysis, 0.2 g of leaf tissue of T1 plants was ground in liquid nitrogen and homogenized in 1 ml PBS (137 mM NaCl, 2.7 mM KCl, 10 mM Na2HPO4, 1.8 mM KH2PO4, pH 7.4) containing 4 mM β-mercaptoethanol to preserve thiol groups and prevent aggregation. Extracts were clarified by centrifugation (21,000 × g, 20 min, 4°C) and separated into soluble (supernatant) and insoluble (pellet) fractions. Two volumes of each fraction were mixed with one volume of 3X Laemmli buffer, heated at 99°C for 10 min. Proteins were separated on 15% polyacrylamide gels, stained with Coomassie Brilliant Blue, or transferred to nitrocellulose membranes, with transfer quality verified by Ponceau S staining. Membranes were blocked with 5% (w/v) non-fat dry milk in 1× TTBS (20 mM Tris-HCl, 150 mM NaCl, 0.1% Tween-20, pH 7.5) and incubated with a rabbit α -hGAL1 IgG (1:7000), followed by an alkaline phosphatase–conjugated secondary antibody (1:2000). Detection was performed in alkaline phosphatase buffer using BCIP/NBT as chromogenic substrates.

### Protein quantification

Soluble protein extracts from T1 plants were obtained from 0.2 g of leaf tissue (fourth leaf from the top) in 1 mL PBS (pH 7.4) containing 4 mM β-mercaptoethanol, centrifuged twice (20,000 × g, 20 min). Total soluble protein (TSP) was determined by BCA assay, and ELISA plates were loaded based on TSP equivalence (1–7 µg TSP/mL for G1 and 14–28 µg TSP/mL for NT samples), using 1X PBS; 0,05% Tween-20; BSA 1% as diluyent and assay blank. Detection was performed as previously described in Croci et al., 2012, using rabbit α-hGAL1 IgG as capture antibody and biotinylated α-hGAL1 IgG followed by HRP–streptavidin/TMB for revelation. Purified recombinant *E. coli* hGAL1 0.5–9 ng/mL was used to generate the standard curve.

### Purification of hGAL 1

Recombinant hGAL1 was purified from soluble protein extracts by lactose-Sepharose affinity capture, exploiting the carbohydrate recognition domain of galectins (Guardia et al., 2014). Preliminary column affinity purification was performed as described (Roldán-Montero et al., 2022). To avoid clogging commonly associated with plant extracts in column-based methods, purification was implemented in batch mode. Soluble fractions extracted from 240 mg of leaf tissue (600 ml) were incubated with 13 ml of lactose-Sepharose resin (Sigma Aldrich) for 1 h at 4°C under agitation. The resin was recovered by centrifugation, washed with 120 ml PBS containing 4 mM β-mercaptoethanol, and eluted with 200 mM lactose (60 ml). Eluates were sterile filtered (0.22 μm) and buffer-exchanged to 1X PBS pH 7,4 containing 4 mM β-mercaptoethanol (0.7-1.3 ml). Similar process was applied to protein extracts of non-transformed plants and to purificate hGal1 from *E. Coli* culture (300ml) after sonication. Fractions were evaluated by SDS-PAGE and Western blot.

### Carbohydrate-binding activity

Glycan-binding activity of hGAL1 was assessed in solid-phase binding assays (Rapoport et al., 2010; Cagnoni et al., 2024). Microplates coated with asialofetuin (ASF) were incubated with purified hGAL1 (from batch mode) or hGal1st in the presence of serial dilutions of lactose (Sigma Aldrich) or N-acetyllactosamine (Elicityl). Bound protein was detected with a biotinylated α-hGAL1 antibody, streptavidin-HRP, and TMB/H_2_O_2_, with absorbance measured at 450 nm. Half maximal inhibitory concentration (IC_50_) values were calculated (mean ± SD, n = 3).

### Induction of T cell apoptosis

The immunoregulatory activity of plastid-derived hGAL1 was evaluated by its ability to induce T cell apoptosis of Briefly. Jurkat T cells were incubated with increasing concentrations of purified hGAL1 (from batch mode) or hGal1st in presence or absence of 20 mM lactose as a competitive inhibitor. As a negative control, an extract obtained from non-transformed plants and purified under identical conditions was included. After 6 h, early and late apoptosis was determined by Annexin V-FITC and propidium iodide staining followed by flow cytometry.

### Statistical analysis

All analyses were performed in R using RStudio. Linear mixed-effects models were applied to ELISA data, and two-way ANOVA to apoptosis assays. Pairwise comparisons were conducted with the emmeans package using Tukey or Sidak adjustment; differences were considered significant at p < 0.05.

## Results

### Generation and molecular characterization of transplastomic lines

The hGAL1 coding sequence was sub cloned into the chloroplast transformation vector pBSWUTR previously developed in our laboratory (Wirth et al., 2006). The transgene was inserted downstream of the promoter and 5’ untranslated region (5´UTR) of the *psbA* gene (Fernández-San Millán et al., 2003; Eibl et al., 1999). The vector pBSWUTR hGAL1 contains a selectable marker gene (*aadA)* that confers spectinomycin resistance to transplastomic shoots. This vector mediates site-specific integration of transgenes into the *rrn* operon of the plastome, in the intergenic region located between the ribosomal *16 s* and the *trnI* genes (**Figure 1A**). Biolistic transformation of tobacco leaves with the pBSWUTR hGAL1 plasmid yielded multiple spectinomycin-resistant shoots after 4–6 weeks of regeneration. Initial PCR screening confirmed transgene integration (**Figure 1B**). G1A, G1B and G1C positive plants were subjected to additional regeneration rounds in spectinomycin-containing medium to obtain homoplasmy. Plants from the third regeneration cycle were transferred to soil and grown to maturity. The phenotypic appearance of the transplastomic and non-transformed plants was indistinguishable after 15 weeks of growth under greenhouse conditions (**Figure 1C**). No differences were observed when comparing growth rate, flowering time and germination rate between the transplastomic lines and non-transformed type plants.

Southern blot analysis was performed to confirm transgene integration and assess the homoplasmy in regenerated lines. Total leaf DNA was extracted, digested with *NcoI* restriction enzyme and separated by electrophoresis. Blot was hybridized with a probe specific for the trnI-trnA region. The expected 1.5 kb fragment size was detected in transformed lines confirming correct site-specific integration of the *hGAL1* cassette (**Figure 1D**). In contrast, NT plants exhibited the diagnostic 6.5 kb band. The absence of the 6.5 hybridization fragment in transplastomic lines indicated the elimination of residual non-transformed plastomes copies following successive regeneration cycles under antibiotic selection.

Homoplasmy was further analyzed by germination assay on MS medium supplemented with spectinomycin. All progeny from G1 lines displayed complete resistance, whereas NT seeds germinated only in the absence of the antibiotic (**Figure 1E**). These results confirmed stable integration of the transgene, reinforcing the conclusion that G1A, G1B, and G1C lines had achieved homoplasmy.

### Analysis of transgene transcription

**Figure 2:**
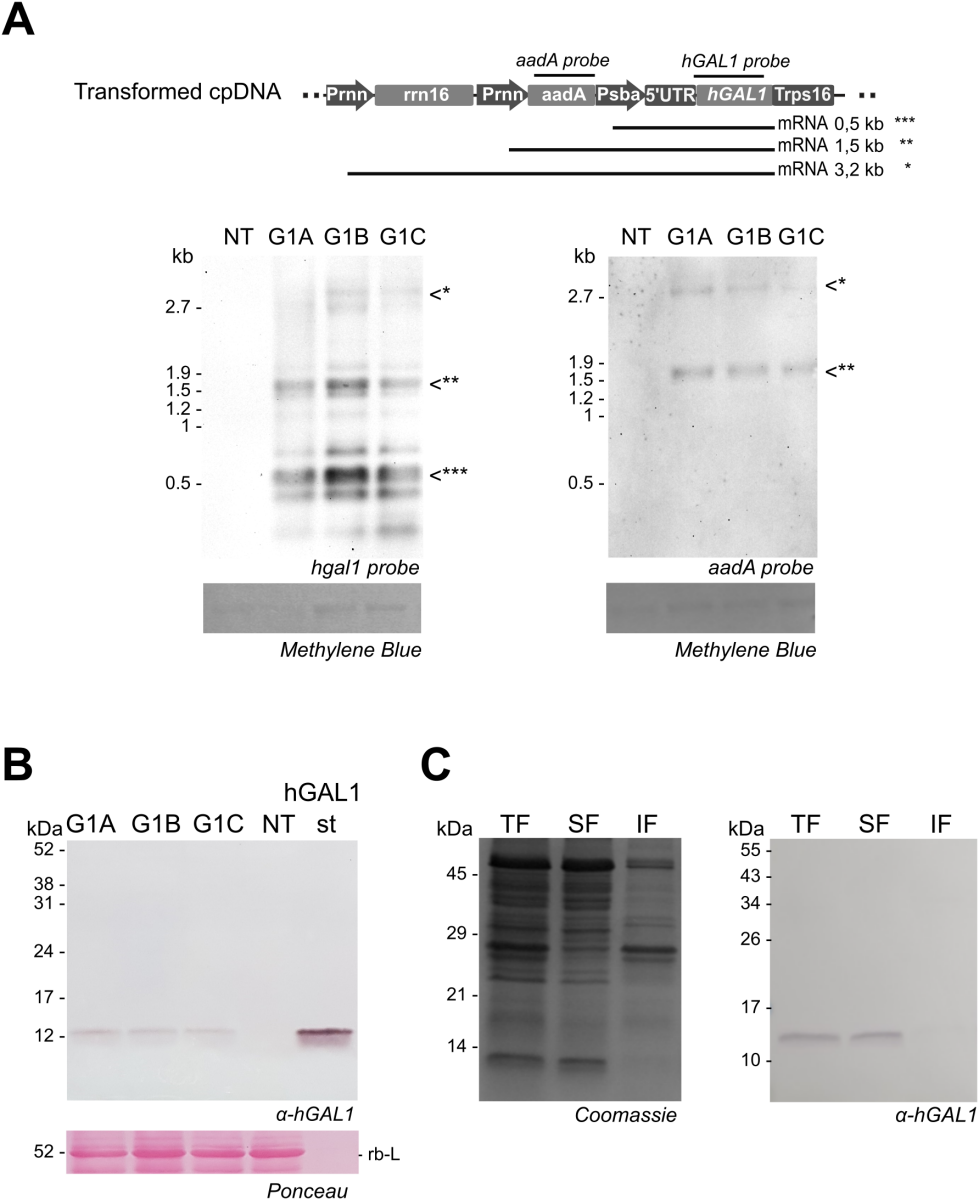
hGAL1 expression in transplastomic lines. **(A)** Northern blot of 1 µg total RNA from G1 A–C and NT plants, hybridized with *hGAL1* (left) and *aadA* (right) probes. Equal RNA loading was verified by methylene blue staining of the ribosomal RNA (rRNA) bands in the membrane. The upper panel shows a schematic representation of the probe locations and the expected mRNA transcripts, indicated as *, **, and *** in the blots. The position of 23S (2.9 kb) and 16S (1.5 kb) rRNA is indicated on the left. **(B)** Western blot analysis of total leaf protein from G1 A–C and NT plants (4.5 mg of leaf tissue) using α-hGAL1 antibodies. RuBisCO large subunit (rb-L) stained with Ponceau S serves as a loading control. hGAL1st: 200 ng of purified recombinant human galectin 1 expressed in *E. coli*. **(C)** Solubility profile of hGAL1 in G1 line (5 mg of leaf tissue) in PBS containing β-mercaptoethanol. The SDS-PAGE gel stained with Coomassie is shown on the left, and an equal Western blot detected with α-hGAL1 antibodies is shown on the right. TF, total fraction; SF, soluble fraction; IF, insoluble fraction.

Transgene transcription in transplastomic lines was assessed by Northern blot. For this purpose, total RNA was extracted from leaves and subjected to electrophoretic separation. Three types of transcripts were observed after hybridization with the *human hGAL1* probe in the transplastomic lines but not in the NT plants. The revealed pattern included monocistronic transcripts corresponding to transgene sequence transcribed from the *psbA* promoter (present in the 5´*psbA* sequence), bicistronic transcripts transcribed from the *rrn* promoter (*Prrn*) included in the cassette, and a larger transcript generated by read-through transcription from the endogenous promoter of the *rrn* operon (**Figure 2A**). The identity of bicistronic and polycistronic transcripts was confirmed by hybridization with the *aadA* probe. The electrophoretic mobility for each transcript was consistent with the expected sizes of the three transplastomic lines analyzed confirming *hGAL1* transgene expression.

### Recombinant hGAL1 accumulation in plants

Recombinant protein accumulation in transplastomic lines was verified by Western blot using a polyclonal antibody against hGAL1. Analysis of total protein extracts from leaf tissue revealed a band of the expected size (~14.5 kDa) in the three transplastomic lines (**Figure 2B**). The absence of additional bands of higher or lower molecular weight suggests that no detectable aggregation, proteolysis, or other post-translational modifications occurred in the recombinant hGAL1 expressed in chloroplasts. Furthermore, hGAL1 expression was analyzed in leaves at different developmental stages, revealing an increase in accumulation correlated with plant age (**Supplementary Figure S1A**). Mature leaves accumulated higher protein levels than younger leaves, and hGAL1 remained stable in senescent tissue. Interestingly, plants grown under dark conditions showed enhanced hGAL1 accumulation (**Supplementary Figure S1B**).

Western blot analysis revealed the expected band of ~14.5 kDa in the total protein sample and the soluble fraction but not in the insoluble fraction confirming that plastid-produced hGAL1 was fully soluble (**Figure 2C**). hGAL1 accumulation was quantified by ELISA.

Transplastomic lines produced soluble hGAL1 at average yield of 5.67 mg per kilogram of leaf tissue corresponding to approximately 0.05% of total soluble protein (TSP) (**Supplementary Figure S2**, **Supplementary Table S1**).

### Batch-mode purification of hGAL1

**Figure 3:**
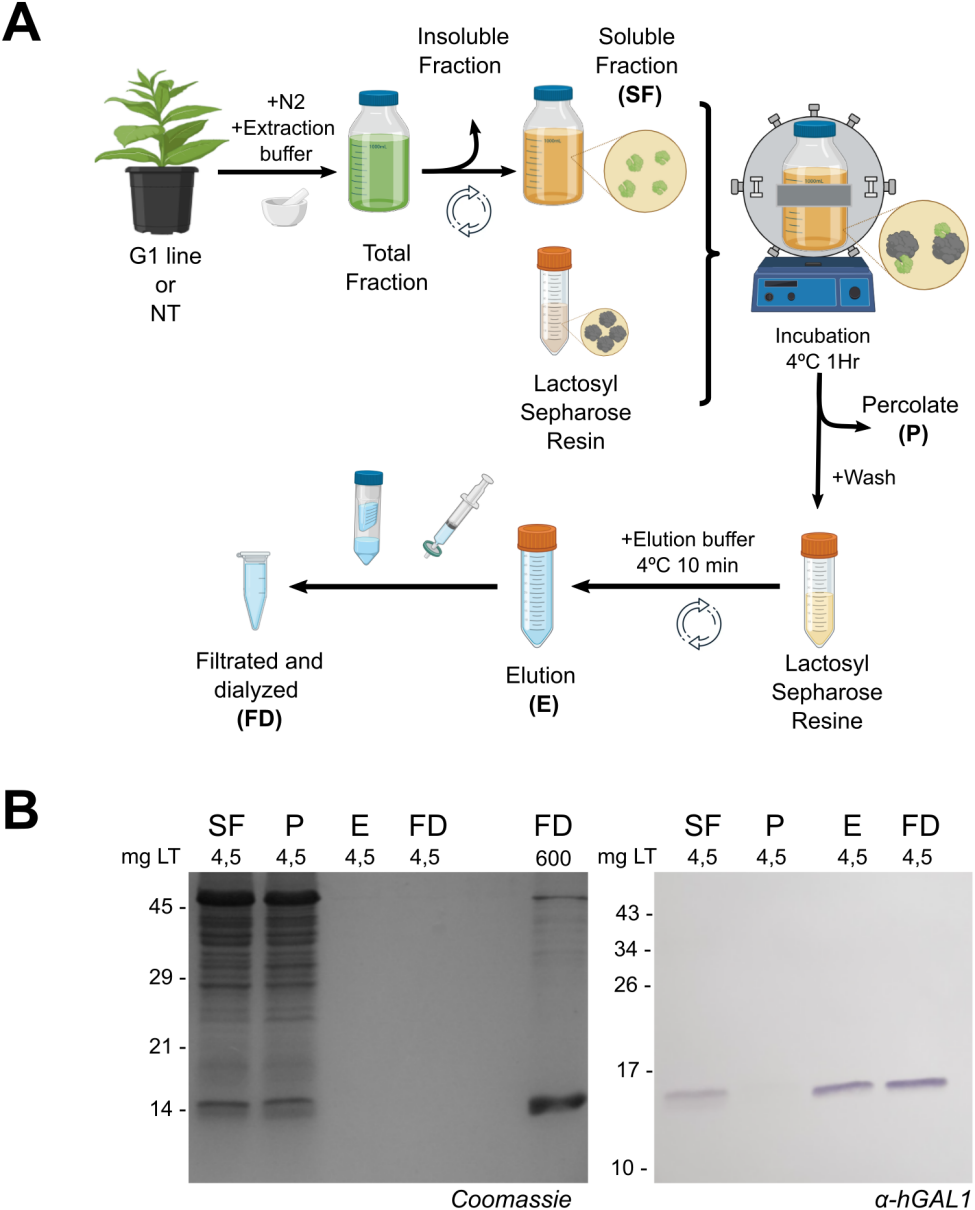
Batch purification. **(A)** Schematic representation of the purification workflow using Lactosyl Sepharose resin, including sequential precipitation and concentration steps. **(B)** Purification profile from 240 g of pooled leaf tissue from G1 plants. Protein fractions analyzed by SDS PAGE stained with Coomassie Blue (left) and equal Western blot using α-hGAL1 antibodies (right). SF, Soluble fraction incubated with the resin; P, percolate; E, elution; FD, filtered and dialyzated fraction. mg LT: leaf tissue mass corresponding to the sample.

Recombinant hGAL1 is commonly purified by α-lactose Sepharose affinity chromatography. To adapt this approach for plant extracts, we developed a simplified purification procedure that avoided resin clogging typically observed during column chromatography. This protocol was implemented in batch mode, to streamline the workflow and reduce downstream costs (**Figure 3A**).

Analysis of the elution fractions by SDS-PAGE followed by Coomassie Blue staining showed a clear enrichment of a protein band corresponding to the expected molecular mass of hGAL1 (~14.5 kDa). Traces of co-eluting proteins were only detectable when the loaded sample was concentrated more than 100-fold (**Figure 3B**). Western blot analysis confirmed that no signal was detected with the α-hGAL1 antibody in the percolate (P), indicating efficient retention of hGAL1 on the affinity matrix. No evidence of proteolysis or higher– or lower–molecular-weight species was observed in the elution fraction or in the final preparation.

The final preparation was sterile and directly suitable for downstream applications. When processing 240 g of leaf tissue, this procedure reached higher yields than those obtained using affinity columns, while reducing purification time (**Supplementary Table S2**). For the functional evaluation assays, the purification procedure was also carried out using extracts from non-transformed plants and from *E. coli* expressing hGAL1 (**Supplementary Figure S3**).

### Biochemical validation: carbohydrate-binding activity of plastid-derived hGAL1

**Figure 4:**
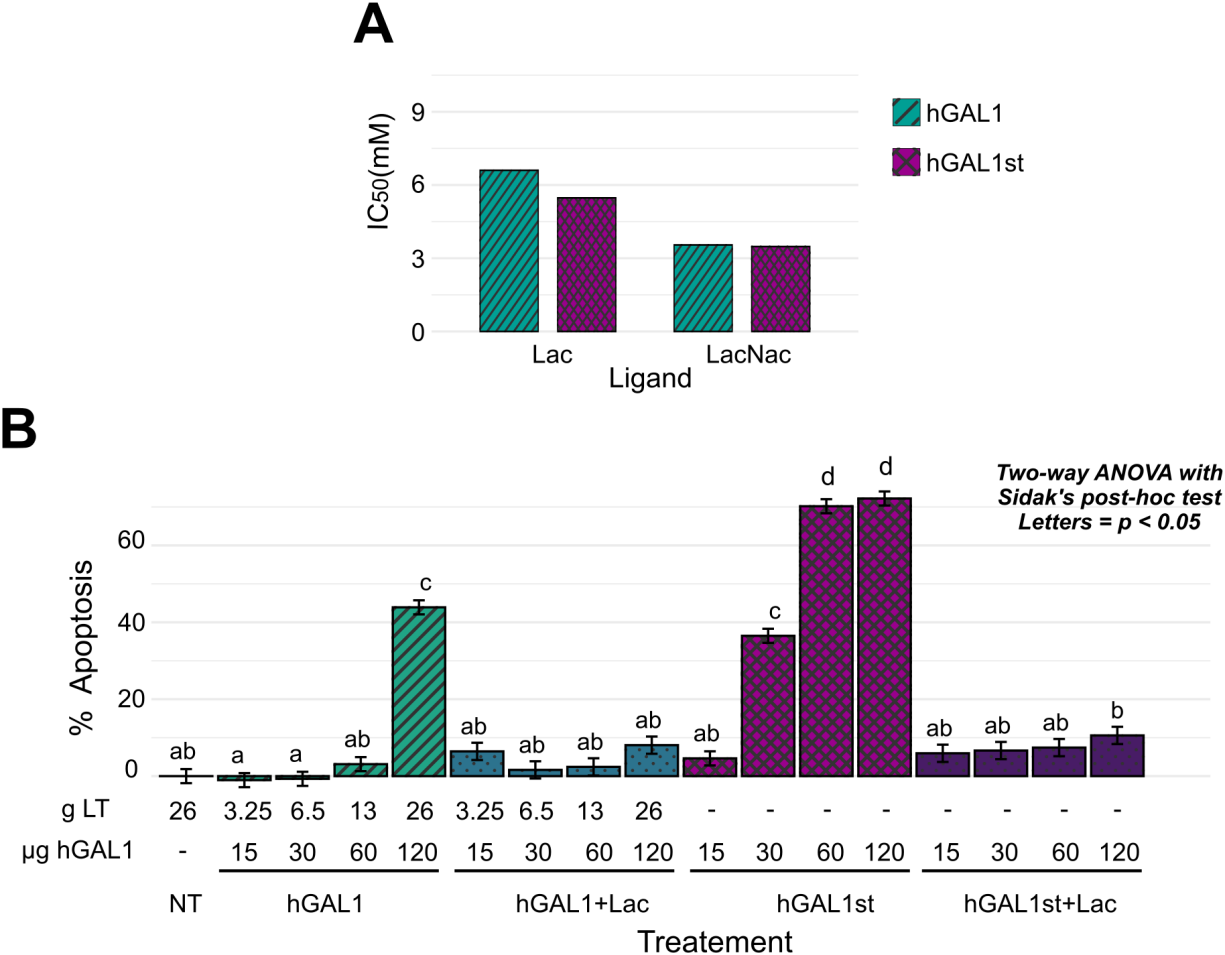
Biological activity of plastid expressed hGAL1. **(A)** Solid-phase binding assay (SPA) assessing hGAL1 affinity for lactose (Lac) and N-acetyllactosamine (LacNac) using asialofetuin (ASF) as ligand. Inhibition curves generated from serial dilutions of Lac or LacNac. Half maximal inhibitory concentration (IC_50_) values (mean, n = 3) are showed for plant-derived (hGAL1) and *E*. *coli* derived hGAL1 (hGAL1st) used as positive control. **(B)** Induction of apoptosis in Jurkat T cells following 6 h exposure to increasing concentrations of hGAL1 (15–120 µg), with or without 20 mM lactose (Lac). Apoptosis was quantified by Annexin V staining and analyzed with a two-factor linear model (treatment × dose) using lm(), followed by estimated marginal means comparisons with the *emmeans* package and Sidak correction; different letters indicate significant differences (*p* < 0.05), n=3. g LT, leaf tissue mass corresponding to the extract used in each treatment; µg hGAL1, mass of purified hGAL1 applied per treatment; NT, extract from non-transformed plants processed under identical purification conditions.

Carbohydrate-binding activity, a prerequisite for the biological function of hGAL1, was evaluated by solid-phase binding assays using asialofetuin (ASF)-coated microplates. Lactose and N-acetyllactosamine were tested as competitive inhibitors of interaction between hGAL1 and ASF, and IC_50_ values were calculated for each compound. The binding profile comparison between plastid hGAL1 and the recombinant hGAL1 from *E. coli* utilized as standard reference hGAL1st, showed no significant difference within both ligands (**Figure 4A**; **Supplementary Table S3**). These findings indicate preservation of the native carbohydrate recognition domain and confirm that chloroplast expression in chloroplasts does not compromise the lectin’s ability to recognize specific glycans.

### Functional validation: induction of apoptosis of Jurkat T cells

To assess the biological activity of the hGAL1 produced in tobacco chloroplasts, its immunomodulatory capacity was evaluated by analyzing apoptosis induction in Jurkat T cells. Cells were incubated with different concentrations of plastid hGAL1 or the standard hGAL1, in the presence or absence of lactose as a competitive inhibitor. Results revealed a dose-dependent induction of apoptosis (**Figure 4B**; **Supplementary Table S4**). Treatment with plastid hGAL1 showed a significant increase in apoptosis only at the highest concentration tested (120 µg hGal1/ml), reaching an average of 48.8 ± 2.2% apoptotic cells. In contrast, bacterial hGAL1 induced apoptosis in a dose-dependent manner, reaching 73 ± 1.3% apoptotic cells at 120 µg hGal1/ml. The pro-apoptotic potency of plant-derived hGAL1 was approximately 70% of that observed for the bacterial counterpart.

Co-incubation with lactose significantly reduced apoptosis induced in both plant- and bacteria-derived hGAL1 at all concentrations tested. In the presence of lactose, apoptosis levels remained below 15%, similar to unstimulated controls. Control purifications from non-transformed plants (**Supplementary Figure S3**) did not show binding activity in the carbohydrate-recognition assays nor pro-apoptotic activity in Jurkat cells (**Figure 4**; **Supplementary Table S4**), indicating that endogenous lectins—if present—did not contribute detectable background signals. These findings confirm the specificity of hGAL1-mediated T cell ted apoptosis, demonstrating that this effect occurred through its carbohydrate-binding activity.

## Discussion

Chloroplast transformation in *Nicotiana tabacum* proved suitable for producing soluble and functional hGAL1. Three aspects were central to this proof-of-concept: stable transgene integration, accumulation of a structurally competent protein, and implementation of a simplified purification workflow compatible with plant extracts.

### Expression and folding environment in plastids

The successful generation of homoplasmic lines that express hGAL1 without evident phenotypic penalties indicates that hGAL1 acumulation imposes minimal metabolic burden on the plastid compartment. In contrast, other proteins expressed in plastids has led to chlorosis or growth delay (Müller et al., 2024; Castiglia et al., 2016). The transcriptional pattern observed by Northern blot analysis revealed the expected transcript diversity—monocistronic, bicistronic, and polycistronic—driven by the *psbA* and *rrn* promoters (Maliga, 2004; Bock, 2015). This profile demonstrates efficient recognition of regulatory plastid elements and hGal1 sequence by plastid transcriptional machinery. Together, these data confirm that the chloroplast provides a compatible environment for hGAL1 expression.

The solubility of plastid-produced hGAL1 is particularly relevant. The absence of soluble aggregates - within the detection limits of our western blot assays- suggests that plastid hGAL1 remained soluble. In contrast, preliminary results from our laboratory showed that hGAL1 transiently expressed in the tobacco apoplast accumulates in an insoluble form. Importantly, the elimination of the need for refolding steps constitutes a major advantage of plastid expression, which provides a favorable folding environment for hGAL1 (Bally et al., 2008).

### Accumulation levels and physiological modulation

Although the accumulation level (~0.05% TSP) is modest relative to the highest-yielding plastid-expressed proteins (De Cosa et al., 2001; Oey et al., 2009), it falls within the broad range reported for transplastomic systems (Ahmad et al., 2016). The observed influence of leaf developmental stage and photoperiod suggests that hGAL1 accumulation is sensitive to plastid redox and metabolic state, mirroring effects previously described for other cysteine-rich plastid-derived proteins (Staub et al., 2000; Wirth et al., 2006; Zhang et al., 2013). This effect is in line with the six cysteine residues in hGAL1, which form three intramolecular disulfide bonds in their oxidized state (Cys2–Cys130, Cys16–Cys88, and Cys42–Cys60) (Guardia et al., 2014), suggesting that plastid redox conditions influence hGAL1 folding and stability, and that cultivation strategies and molecular approaches can be optimized to improve yields.

### Purification constraints and simplified batch-mode recovery

Downstream processing is a major bottleneck in plant molecular farming (Buyel, 2019; Shanmugaraj et al., 2020). Although α-lactose–Sepharose affinity chromatography is standard for galectin purification (Dey et al., 2023), plant extracts often hinder column flow due to clogging and non-specific interactions. Here we show that a simplified batch-mode procedure enables efficient recovery of functional hGAL1 while avoiding filtration problems and reducing processing time compared with column formats.

Because plant tissues contain diverse soluble proteins, we evaluated whether endogenous β-galactoside-binding proteins could co-elute with hGAL1. However, control purifications from non-transformed plants displayed neither glycan-binding signals nor apoptotic activity indicating that endogenous lectins—if present—did not contribute to detectable background signals. The resulting eluates, although not fully purified, were highly enriched in hGAL1, sterile, and suitable for downstream analyses. This streamlined workflow fits with ongoing efforts to develop cost-effective, chromatography-light purification strategies (Buyel, 2019; Shanmugaraj et al., 2020). Recovering functional hGAL1 in a single affinity step strengthens the economic feasibility of plastid-based systems. Future work should aim to integrate batch capture with scalable unit operations to progress toward production-grade purification.

### Functional activity and comparison with bacterial hGAL1

Plastid-derived hGAL1 retained glycan-binding specificity and induced specific apoptosis in T cells, confirming functional integrity. However it showed reduced pro-apoptotic potency relative to *E. coli*-derived hGAL1st. A plausible explanation for the reduced pro-apoptotic potency of plastid-derived hGAL1 is the intrinsic redox sensitivity of this lectin (Rabinovich et al., 1999; Toscano et al., 2006). hGAL1 requires the correct formation of intrachain disulfide bonds and an appropriate monomer–dimer equilibrium to reach full biological activity (Stowell et al., 2009). These parameters are strongly influenced by protein concentration. In our plant extracts, hGAL1 is present at relatively low concentrations (~0.05% TSP), where partial oxidation or shifts in the dimerization state are more likely to occur during extraction or handling. In contrast, *E. coli* expression yields substantially higher protein concentrations, which favor structural stability and preserve dimeric active species. These differences in concentration-dependent stability provide a mechanistic rationale for the higher doses required to elicit apoptosis with the plastid-derived preparation, without implying a loss of intrinsic carbohydrate-binding specificity or folding competence.

### Implications and future directions

The ability to obtain active hGAL1 directly from leaf tissue broadens the toolkit of plastid-produced human proteins. Given the therapeutic potential of hGAL1 therapeutic potential of hGAL1 in inflammation and autoimmunity (Toscano et al., 2006; Rabinovich et al., 2025; Corrêa et al., 2017), the plastid platform may help overcome persistent challenges in producing stable, active lectins at scale. Future work should focus on increasing accumulation via codon optimization, alternative UTRs, fusion tags or relocalization (Morgenfeld et al., 2014; De Marchis et al., 2012), improving stability through optimized extraction and storage conditions, and integrating batch affinity with other low-cost purification technologies. Ultimately, validation in animal models will be required to determine equivalence to bacterially produced protein.

### Concluding remarks

In summary, plastid transformation enables production of soluble, correctly folded hGAL1 and supports a simplified recovery process yielding functional protein. While yields and potency remain below those of *E. coli*, plastids offer unique advantages in biosafety, folding environment, and scalability. With further optimization and process engineering, plastid biotechnology may provide an economically competitive route for producing hGAL1 and related therapeutic lectins.

## Data Availability

The original contributions presented in the study are included in the article/**Supplementary Material**. Further inquiries can be directed to the corresponding author.

## Supplementary material

Supplementary Figure 1 Optimization of hGAL1 expression in transplastomic plants. **(A)** Effect of leaf age on hGAL1 accumulation G1 transplastomic plants. Leaves were numbered from top (1) to bottom (8). Left panel: total protein extracts from leaves 1–8. Right panel: total (TF), soluble (SF), and insoluble (IF) protein fractions from leaves 3, 5, and 8. Total protein was stained with Ponceau Red. Non-transformed (NT) plant extract was use as negative control. **(B)** Effect of photoperiod on hGAL1 accumulation. Left panel: total protein extracts from G1 plants sampled at 2 and 5 days. Middle panel: fractionation into TF, SF, and IF in PBS containing β-mercaptoethanol. Right panel: independent experiment sampled at 2, 5, 8, and 12 days. At each time point, tissue was collected from the same fourth leaf from T1 G1 plants. Total protein extract obtained from 4 mg of plant tissue was loaded per lane. Bar graphs show relative hGAL1 abundance (mean ± SE), calculated as the ratio of hGAL1 to RbcL band intensity from three independent replicates quantified with ImageJ. Loading control: RbcL stained with Ponceau Red. L/D, 16 h light/8 h dark; Dark, continuous darkness; d, days of treatment.

Supplementary Figure 2 Determination of hGAL1 accumulation in transplastomic tobacco plants. hGAL1 in soluble protein extracts from G1 lines (fourth leaf from the top) was measured by ELISA using rabbit α-hGAL1 antibodies and recombinant *E. coli* hGAL1 as standard. Acumulation expressed as mg hGAL1 per kilogram of leaf tissue (mg hGAL1/kg LT, upper panel) and %hGAL1/TSP (lower panel). Data were analyzed in R using a linear mixed-effects model on log-transformed values (upper panel) and a beta model (lower panel), with Line (G1 A, B, C) as a fixed effect and assay day (1–4) as a random effect. Pairwise comparisons were performed with the emmeans package and Tukey adjustment; estimated marginal means (back-transformed) are shown ± 95% CI. NT plants showed no detectable signal. Data represent four independent ELISA assays (n = 4), each including four plants (NT, G1A, G1B, and G1C). For each plant, hGAL1 accumulation was estimated from 3–4 extract concentrations measured in duplicate.

Supplementary Figure 3 Control purifications from *E. coli* and non-transformed (NT) plants. *E. coli* hGAL1 corresponds to recombinant hGAL1 expressed in *E. coli* and purified by lactose-affinity chromatography, used as positive control in biological assays. The NT profile represents the same purification protocol performed from non-transformed *N. tabacum* extract, included as a negative control in apoptosis assay. Fractions were analyzed by SDS-PAGE (Coomassie staining) and Western blot (α-hGAL1). SF, soluble fraction; P, percolate; E, elution; FD, filtered and dialyzed fraction.

Supplementary Table 1 hGAL1 accumulation in transplastomic tobacco lines G1 A-C measured by ELISA from soluble protein extracts. The table shows the assay day (Day), transplastomic line (Line), hGAL1 concentration expressed as mg hGAL1 per kilogram of leaf tissue (mg/kg LT), and as percentage of total soluble protein (%hGAL1/TSP). Data corresponds to those presented in **Supplementary Figure S2**.

Supplementary Table 2 Values of purification yields of hGAL1 from transplastomic tobacco plants. The table summarizes the amount of leaf tissue processed, the final extract volume, the concentration of hGAL1 in the soluble fraction, the recovered hGAL1 per kilogram of leaf tissue, and the overall purification yield (%), calculated as the mass of purified hGAL1 recovered at the end of the purification process relative to the total hGAL1 content in the corresponding soluble extract at the start of the purification process. Mean and standard deviation (SD) values are reported.

Supplementary Table 3 IC_50_ values from solid-phase binding assay (SPA) shown in **Figure 4A**, assessing hGAL1 affinity for lactose (Lac) and N-acetyllactosamine (LacNAc). Columns indicate the ligand, protein, and corresponding IC_50_ value. hGAL1st refers to recombinant human Galectin 1 expressed in *E. coli* as standard reference.

Supplementary Table 4 Values from the apoptosis induction experiment shown in **Figure 4B**, in Jurkat T cells treated with plastid hGAL1 and the standard hGAL1st, in the absence or presence of lactose (Lac). Columns indicate treatment, protein dosage (µg hGAL1), equivalent fresh leaf tissue (g LT), and percentage of apoptosis (%). %Apoptosis was calculated from Annexin V/PI staining as (%Annexin V^+^PI^+^ in treated – %Annexin V^+^PI^+^ in untreated)/(%Annexin V^-^PI^-^ in untreated) × 100.
