# Supplementary figures and images for: Production of functional human galectin-1 in transplastomic tobacco and simplified recovery via batch-mode purification
Source: Front Plant Sci. 2026 Jan 2;16:1721928. doi: 10.3389/fpls.2025.1721928 (PMC12808362; doi:10.3389/fpls.2025.1721928)

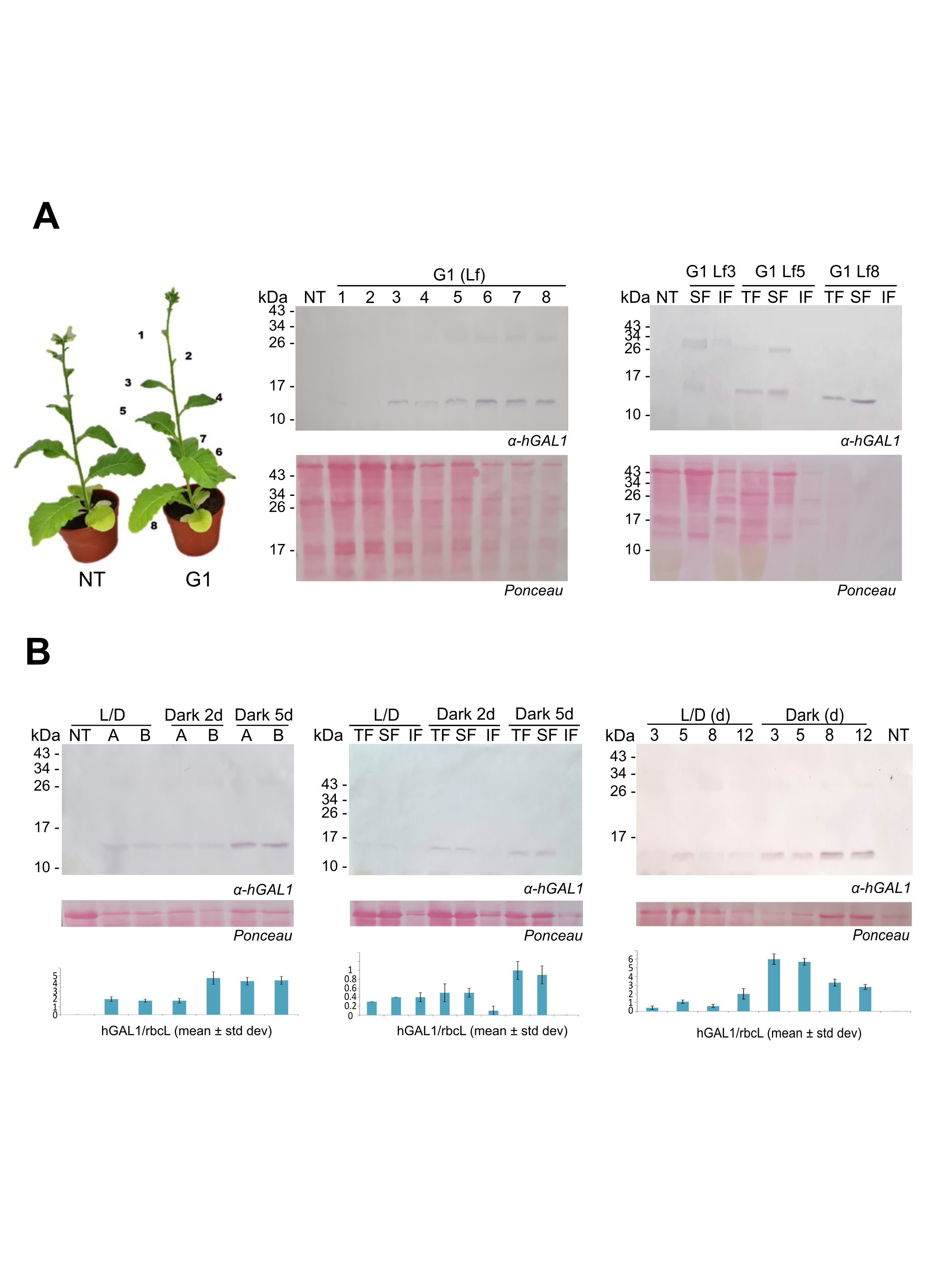

Supplement: Supplementary Figure 1 — Optimization of hGAL1 expression in transplastomic plants. (A) Effect of leaf age on hGAL1 accumulation G1 transplastomic plants. Leaves were numbered from top (1) to bottom (8). Left panel: total protein extracts from leaves 1–8. Right panel: total (TF), soluble (SF), and insoluble (IF) protein fractions from leaves 3, 5, and 8. Total protein was stained with Ponceau Red. Non-transformed (NT) plant extract was use as negative control. (B) Effect of photoperiod on hGAL1 accumulation. Left panel: total protein extracts from G1 plants sampled at 2 and 5 days. Middle panel: fractionation into TF, SF, and IF in PBS containing β-mercaptoethanol. Right panel: independent experiment sampled at 2, 5, 8, and 12 days. At each time point, tissue was collected from the same fourth leaf from T1 G1 plants. Total protein extract obtained from 4 mg of plant tissue was loaded per lane. Bar graphs show relative hGAL1 abundance (mean ± SE), calculated as the ratio of hGAL1 to RbcL band intensity from three independent replicates quantified with ImageJ. Loading control: RbcL stained with Ponceau Red. L/D, 16 h light/8 h dark; Dark, continuous darkness; d, days of treatment. [file Image1.tiff]

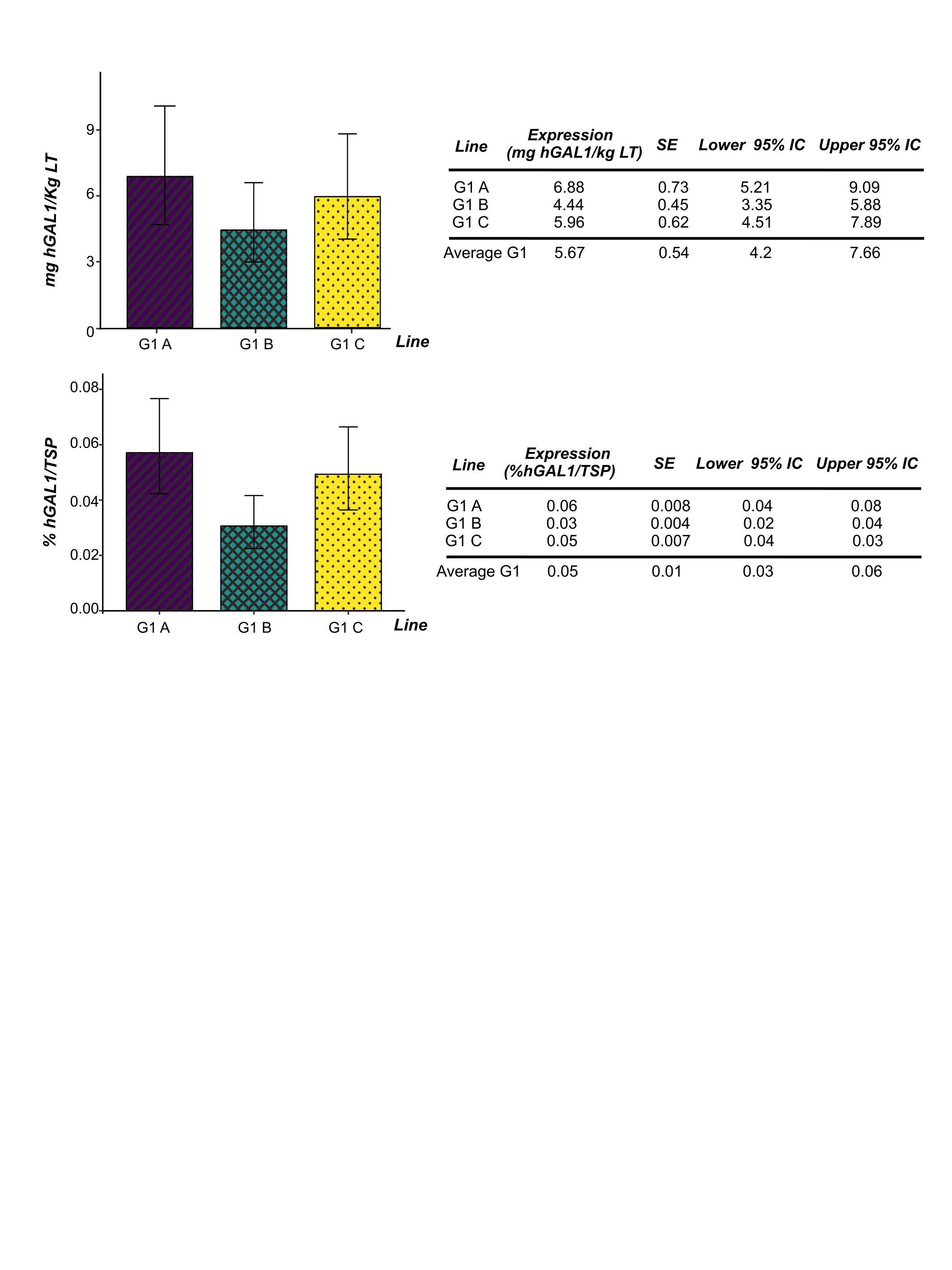

Supplement: Supplementary Figure 2 — Determination of hGAL1 accumulation in transplastomic tobacco plants. hGAL1 in soluble protein extracts from G1 lines (fourth leaf from the top) was measured by ELISA using rabbit α-hGAL1 antibodies and recombinant E. coli hGAL1 as standard. Acumulation expressed as mg hGAL1 per kilogram of leaf tissue (mg hGAL1/kg LT, upper panel) and %hGAL1/TSP (lower panel). Data were analyzed in R using a linear mixed-effects model on log-transformed values (upper panel) and a beta model (lower panel), with Line (G1 A, B, C) as a fixed effect and assay day (1–4) as a random effect. Pairwise comparisons were performed with the emmeans package and Tukey adjustment; estimated marginal means (back-transformed) are shown ± 95% CI. NT plants showed no detectable signal. Data represent four independent ELISA assays (n = 4), each including four plants (NT, G1A, G1B, and G1C). For each plant, hGAL1 accumulation was estimated from 3–4 extract concentrations measured in duplicate. [file Image2.tiff]

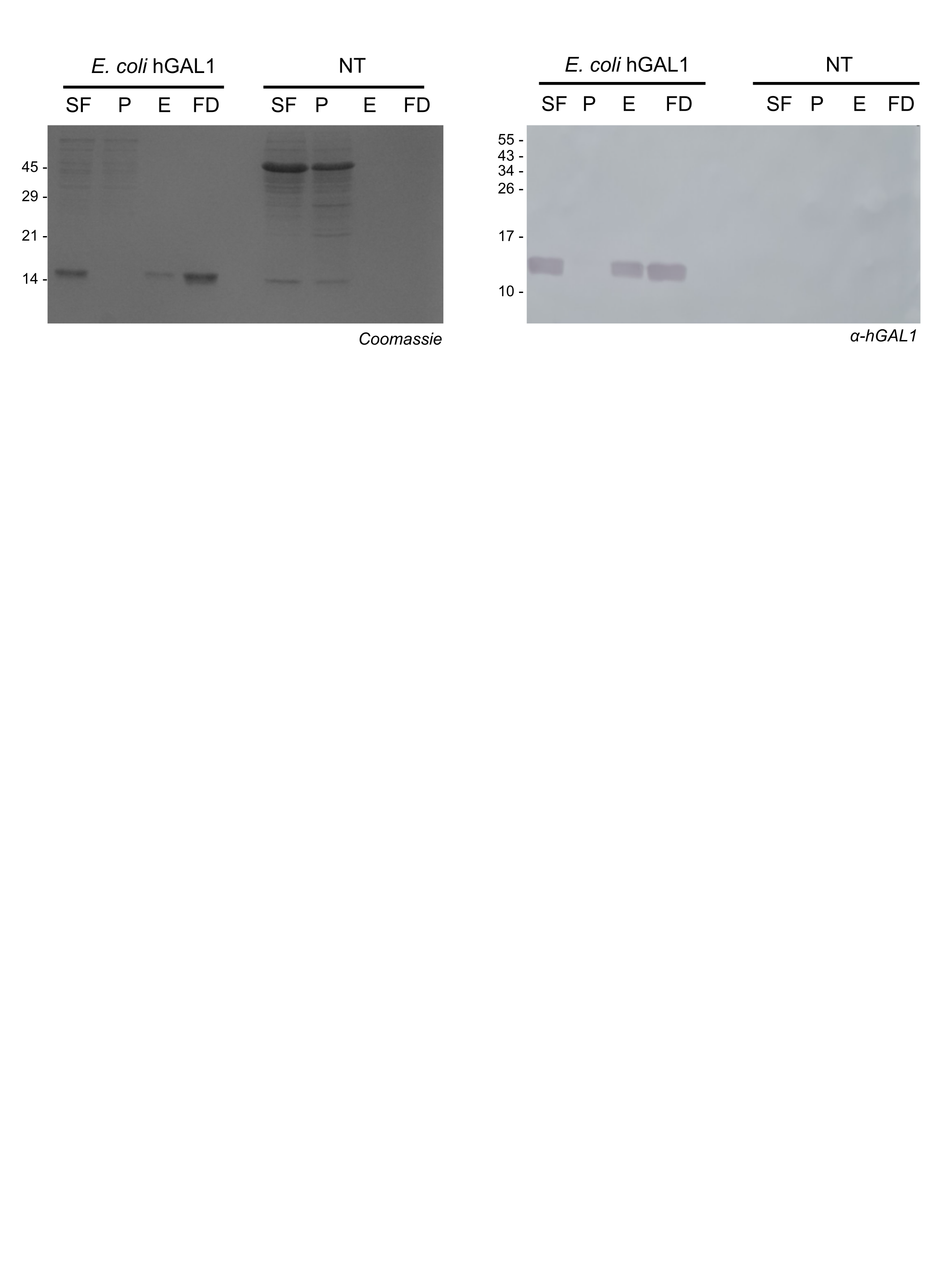

Supplement: Supplementary Figure 3 — Control purifications from E. coli and non-transformed (NT) plants. E. coli hGAL1 corresponds to recombinant hGAL1 expressed in E. coli and purified by lactose-affinity chromatography, used as positive control in biological assays. The NT profile represents the same purification protocol performed from non-transformed N. tabacum extract, included as a negative control in apoptosis assay. Fractions were analyzed by SDS-PAGE (Coomassie staining) and Western blot (α-hGAL1). SF, soluble fraction; P, percolate; E, elution; FD, filtered and dialyzed fraction. [file Image3.tiff]
