# Supplementary material for: Production of functional human galectin-1 in transplastomic tobacco and simplified recovery via batch-mode purification
Source: Front Plant Sci. 2026 Jan 2;16:1721928. doi: 10.3389/fpls.2025.1721928 (PMC12808362; doi:10.3389/fpls.2025.1721928)
Supplement: Supplementary Table 1 — hGAL1 accumulation in transplastomic tobacco lines G1 A-C measured by ELISA from soluble protein extracts. The table shows the assay day (Day), transplastomic line (Line), hGAL1 concentration expressed as mg hGAL1 per kilogram of leaf tissue (mg/kg LT), and as percentage of total soluble protein (%hGAL1/TSP). Data corresponds to those presented in Supplementary Figure S2. [file Table1.docx]

| Day | Line | mg GAL1/Kg LF | %hGAL1/TSP |
| --- | --- | --- | --- |
| 1 | G1 A | 5.907 | 0.040 |
| 1 | G1 A | 6.180 | 0.042 |
| 1 | G1 A | 6.923 | 0.047 |
| 1 | G1 B | 4.355 | 0.025 |
| 1 | G1 B | 4.570 | 0.026 |
| 1 | G1 B | 5.565 | 0.032 |
| 1 | G1 C | 4.906 | 0.034 |
| 1 | G1 C | 5.365 | 0.038 |
| 1 | G1 C | 6.464 | 0.045 |
| 1 | NT | 0.038 | 0.000 |
| 1 | NT | 0.045 | 0.000 |
| 1 | NT | 0.068 | 0.000 |
| 2 | G1 A | 4.684 | 0.044. |
| 2 | G1 A | 4.830 | 0.046 |
| 2 | G1 A | 5.775 | 0.054 |
| 2 | G1 A | 6.613 | 0.064 |
| 2 | G1 B | 3.376 | 0.027 |
| 2 | G1 B | 2.939 | 0.024 |
| 2 | G1 B | 2.932 | 0.024 |
| 2 | G1 C | 6.198 | 0.042 |
| 2 | G1 C | 6.146 | 0.042 |
| 2 | G1 C | 6.705 | 0.046 |
| 2 | G1 C | 6.519 | 0.046 |
| 2 | NT | 0.114 | 0.000 |
| 2 | NT | 0.087 | 0.000 |
| 2 | NT | 0.002 | 0.000 |
| 3 | G1 A | 8.153 | 0.056 |
| 3 | G1 A | 9.223 | 0.063 |
| 3 | G1 B | 3.626 | 0.038 |
| 3 | G1 B | 3.983 | 0.042 |
| 3 | G1 B | 4.698 | 0.049 |
| 3 | G1 B | 4.968 | 0.053 |
| 3 | G1 C | 3.974 | 0.054 |
| 3 | G1 C | 4.215 | 0.058 |
| 3 | G1 C | 4.769 | 0.065 |
| 3 | G1 C | 5.292 | 0.074 |
| 3 | NT | 0.032 | 0.000 |
| 3 | NT | 0.050 | 0.001 |
| 3 | NT | 0.128 | 0.002 |
| 4 | G1 A | 7.880 | 0.050 |
| 4 | G1 A | 9.196 | 0.059 |
| 4 | G1 B | 5.746 | 0.029 |
| 4 | G1 B | 5.581 | 0.028 |
| 4 | G1 B | 6.286 | 0.028 |
| 4 | G1 B | 6.311 | 0.031 |
| 4 | G1 C | 7.776 | 0.047 |
| 4 | G1 C | 8.210 | 0.054 |
| 4 | NT | 0.071 | 0.000 |
| 4 | NT | 0.059 | 0.000 |
| 4 | NT | 0.110 | 0.000 |
