# Supplementary material for: Production of functional human galectin-1 in transplastomic tobacco and simplified recovery via batch-mode purification
Source: Front Plant Sci. 2026 Jan 2;16:1721928. doi: 10.3389/fpls.2025.1721928 (PMC12808362; doi:10.3389/fpls.2025.1721928)
Supplement: Supplementary Table 2 — Values of purification yields of hGAL1 from transplastomic tobacco plants. The table summarizes the amount of leaf tissue processed, the final extract volume, the concentration of hGAL1 in the soluble fraction, the recovered hGAL1 per kilogram of leaf tissue, and the overall purification yield (%), calculated as the mass of purified hGAL1 recovered at the end of the purification process relative to the total hGAL1 content in the corresponding soluble extract at the start of the purification process. Mean and standard deviation (SD) values are reported. [file Table2.docx]

| Protocol | Leaf tissue processed (g) | Final volume (ml) | hGAL1 (mg/ml) | hGAL1 (mg/kg LT) | Purification yield (% hGAL1 purified/ total) | Mean (%) | SD (%) |
| --- | --- | --- | --- | --- | --- | --- | --- |
| Column | 240 | 30 | 0.03 | 3.750 | 66.14 | 69.81 | 5.20 |
| Column | 240 | 20 | 0.05 | 4.167 | 73.49 |  |  |
| Batch | 240 | 1.3 | 0.9 | 4.875 | 84.05 | 80.53 | 4.52 |
| Batch | 240 | 0.7 | 1.5 | 4.375 | 75.43 |  |  |
| Batch | 240 | 0.7 | 0.8 | 4.762 | 82.1 |  |  |
