# Supplementary material for: Production of functional human galectin-1 in transplastomic tobacco and simplified recovery via batch-mode purification
Source: Front Plant Sci. 2026 Jan 2;16:1721928. doi: 10.3389/fpls.2025.1721928 (PMC12808362; doi:10.3389/fpls.2025.1721928)
Supplement: Supplementary Table 3 — IC50 values from solid-phase binding assay (SPA) shown in Figure 4A, assessing hGAL1 affinity for lactose (Lac) and N-acetyllactosamine (LacNAc). Columns indicate the ligand, protein, and corresponding IC50 value. hGAL1st refers to recombinant human Galectin 1 expressed in E. coli as standard reference. [file Table3.docx]

| Ligand | Protein | IC50 |
| --- | --- | --- |
| Lac | hGAL1 | 8.211 |
| Lac | hGAL1 | 2.500 |
| Lac | hGAL1 | 5.707 |
| LacNac | hGAL1 | 2.087 |
| LacNac | hGAL1 | 4.870 |
| LacNac | hGAL1 | 15.180 |
| Lac | hGALst | 11.300 |
| Lac | hGALst | 2.303 |
| Lac | hGALst | 6.204 |
| LacNac | hGALst | 1.322 |
| LacNac | hGALst | 5.767 |
| LacNac | hGALst | 17.540 |
