# Supplementary material for: Production of functional human galectin-1 in transplastomic tobacco and simplified recovery via batch-mode purification
Source: Front Plant Sci. 2026 Jan 2;16:1721928. doi: 10.3389/fpls.2025.1721928 (PMC12808362; doi:10.3389/fpls.2025.1721928)
Supplement: Supplementary Table 4 — Values from the apoptosis induction experiment shown in Figure 4B, in Jurkat T cells treated with plastid hGAL1 and the standard hGAL1st, in the absence or presence of lactose (Lac). Columns indicate treatment, protein dosage (µg hGAL1), equivalent fresh leaf tissue (g LT), and percentage of apoptosis (%). %Apoptosis was calculated from Annexin V/PI staining as (%Annexin V+PI+ in treated – %Annexin V+PI+ in untreated)/(%Annexin V-PI- in untreated) × 100. [file Table4.docx]

| Treatment | Dosage  (μg hGAL1) | Leaf Tissue  (g LT) | %Apoptosis |
| --- | --- | --- | --- |
| NT | - | 26 | 0.16 |
| NT | - | 26 | -0.27 |
| NT | - | 26 | 0.10 |
| hGAL1 | 15 | 3.25 | -1.01 |
| hGAL1 | 15 | 3.25 | -1.12 |
| hGAL1 | 15 | 3.25 | -1.00 |
| hGAL1 | 30 | 6.5 | -0.95 |
| hGAL1 | 30 | 6.5 | -0,53 |
| hGAL1 | 30 | 6.5 | -0,62 |
| hGAL1 | 60 | 13 | 2.99 |
| hGAL1 | 60 | 13 | 4.40 |
| hGAL1 | 60 | 13 | 1.95 |
| hGAL1 | 120 | 26 | 33.99 |
| hGAL1 | 120 | 26 | 48.69 |
| hGAL1 | 120 | 26 | 49.00 |
| hGAL1+Lac | 15 | 3.25 | 5,34 |
| hGAL1+Lac | 15 | 3.25 | 7.53 |
| hGAL1+Lac | 30 | 6.5 | 2.58 |
| hGAL1+Lac | 30 | 6.5 | 0.66 |
| hGAL1+Lac | 60 | 13 | 5.22 |
| hGAL1+Lac | 60 | 13 | -0.41 |
| hGAL1+Lac | 120 | 26 | 6.98 |
| hGAL1+Lac | 120 | 26 | 9.13 |
| hGAL1st | 15 | - | 5.19 |
| hGAL1st | 15 | - | 4.61 |
| hGAL1st | 15 | - | 4.05 |
| hGAL1st | 30 | - | 39.58 |
| hGAL1st | 30 | - | 30.16 |
| hGAL1st | 30 | - | 39.72 |
| hGAL1st | 60 | - | 69.88 |
| hGAL1st. | 60 | - | 70.08 |
| hGAL1st | 60 | - | 70.59 |
| hGAL1st | 120 | - | 70.18 |
| hGAL1st | 120 | - | 71.11 |
| hGAL1st | 120 | - | 75.30 |
| hGAL1st+Lac | 15 | - | 6.58 |
| hGAL1st+Lac | 15 | - | 5.30 |
| hGAL1st+Lac | 30 | - | 6.24 |
| hGAL1st+Lac | 30 | - | 7.06 |
| hGAL1st+Lac | 60 | - | 9.85 |
| hGAL1st+Lac | 60 | - | 4.99 |
| hGAL1st+Lac | 120 | - | 10.30 |
| hGAL1st+Lac | 120 | - | 10.87 |
